# A Text-Book of Medicine for Students and Beginners

**Published:** 1887-05

**Authors:** 


A TEXT-BOOK OF MEDICINE FOR STUDENTS AND PRACTITIONERS. *By* DR. ADOLF STRUMPELL, *Formerly Professor and Director of the Medical Policlinie at the University of Leipsic. Translated by permission from the second and third German editions, by* HERMAN F. VICKERY, A.B., M.D., AND PHILIP COOMBS KNAPP, A.M., M.D., *with editorial notes by* FREDERICK C. SHATTUCK, A. M., M.D., *Visiting Physician to the Massachusetts General Hospital, &c., &c. Pp. xx and 981. New York:* D. APPLETON & CO. *Chicago:* A. C. MCCLURG & CO.

The editor of this work has added such notes, enclosed in brackets, as are necessary to adapt it to the use of American students and practitioners. These additions are of two kinds,—the addition of complete chapters upon diseases which, though common in America, do not occur in Germany, as Dengue for example, and such additions to the author's treatment of diseases, and description of health resorts as are needed to represent American ideas upon these subjects. This work has been very acceptably done. The lack of a copy of the German edition prevents an expression of opinion in regard to the accuracy of the translation, with which, in other respects, no fault is to be found. It is, however, a mistake that the magnifying power, in diameters, is not given in connection with the cuts of microscopical preparations.

As a treatise on the practice of medicine the book is relatively strongest in its treatment of diseases of the nervous system, but it is everywhere full. concise and clear. In comparison with Eichhorst's "Patologie und Therapie" it is less complete in its discussion of the subjects treated, and very much less complete in its reference to the literature involved. In a general way it may be said that Strümpell's bears about the same relation to Eichhorst's, that Bartholow's does to Flint's "Practice of Medicine.' The book has rapidly reached its third edition in Germany, and richly deserves its popularity.

